# Automated loop invariant inference through learning-guided program structure analysis

**DOI:** 10.1371/journal.pone.0360726

**Published:** 2026-10-27

**Authors:** Abeer S. Hadad, Jiangbin Zheng, Adeeb A. Ahmed, Fahman Saeed, Mohammad Alkhatib, Safa Hussein Mohammed

**Affiliations:** 1 School of Software, Northwestern Polytechnical University, Xi’an, Shaanxi, China; 2 School of Electro-Mechanical Engineering, Xidian University, Xi’an, Shaanxi, China; 3 College of Computer and Information Sciences, Imam Mohammad Ibn Saud Islamic University (IMSIU), Riyadh, Saudi Arabia; 4 School of Electronics and Information, Northwestern Polytechnical University, Xi’an, Shaanxi, China; University of New England School of Science and Technology, AUSTRALIA

## Abstract

Automated loop invariant inference remains a fundamental challenge in formal verification due to the inherently unbounded search space of candidate invariants. Existing approaches either incur substantial computational overhead during invariant synthesis or rely on manually predefined templates, limiting both scalability and automation. Although recent learning-based methods have shown promise, they often fail to fully exploit the rich structural and semantic information embedded within program representations. This paper presents a learning-guided framework for fully automated loop invariant inference based on a systematic analysis of program structure and semantics. The framework performs a joint analysis of syntactic constructs, control-flow structure, and data-dependence relationships to determine invariant structure before instantiation. These complementary representations are embedded and integrated within a learning-based model that identifies structurally and semantically features and infers expressive invariant templates directly from program structure. The inferred templates are instantiated using execution-derived values, yielding constraint systems that are solved and formally validated using a Satisfiability Modulo Theories solver. Experimental evaluation demonstrates that the proposed approach achieves competitive invariant inference performance on challenging benchmarks. By integrating multi-representation program analysis, the framework effectively supports a wide range of loop structures and advances the state of the art in scalable, expressive, and fully automated loop invariant inference.

## 1 Introduction

Loop invariants are crucial for ensuring the correctness and reliability of software, particularly in safety-critical systems such as aviation, medical devices, and automotive control systems [1,2]. A loop invariant is a property that remains true before and after each iteration of a loop [3], serving as a fundamental component in formal program verification. By maintaining the program’s state consistency throughout execution, invariants help prevent errors and demonstrate that the program behaves correctly. Overall, invariants play a vital role in meeting safety standards and ensuring system correctness and reliability [4].

A wide range of techniques have been proposed for automatic loop invariant inference. Static approaches derive invariants using symbolic reasoning, abstract interpretation, or interpolation-based theorem proving [5–8], but are primarily effective for linear arithmetic and scale insufficiently to nonlinear or complex loop behaviors. Dynamic approaches, such as Daikon [9–11], infer invariants from execution traces by identifying relationships that hold across observed runs. However, they often require predefined invariant templates to infer complex nonlinear relationships and thus struggle to generalize invariants whose underlying structure cannot be directly observed from sampled executions alone. Hybrid guess-and-check methods [12–16] combine candidate generation with formal verification, yet remain constrained by predefined templates or the exploration of rapidly growing candidate spaces. More recently, learning-based approaches have attempted to automate invariant inference using data-driven techniques. Some methods rely on execution traces and counterexample feedback [17–20], while others exploit syntactic features of program source code using reinforcement learning–based models [16,21]. Despite their differences, these approaches typically rely on either dynamic observations or partial loop representations, without explicitly modeling the complementary structural and dependency information necessary for capturing complex variable relationships.

Consider the following C loop that implements integer division through repeated subtraction:

//assert (x>0 && y>0)

int q = 0;

int r = x;

while (r>= y) {

r = r - y;

q = q + 1;

}

// assert(0 <= r < y)

This example illustrates the limitations of relying solely on execution traces or partial structural representations. Trace-based approaches often fail to infer the invariant x=q·y+r because the multiplicative relationship between *q* and *y* cannot be reliably generalized from sampled executions alone and typically requires predefined invariant templates. Similarly, representations such as the Abstract Syntax Tree (AST) can identify the subtraction operation r=r−y and the increment *q* = *q* + 1, but do not explicitly capture the data dependencies that relate these updates across loop iterations. In this loop, the value of *r* is initialized from *x* and repeatedly updated using *y*, while *q* evolves synchronously within the same loop body. Considering the data dependencies together with the AST provides complementary structural information that helps reveal the relationship among *q*, *y*, *x*, and *r*, which is essential for inferring the invariant.

This paper presents a deep learning–based framework for loop invariant inference that integrates multiple complementary representations to capture a comprehensive view of loop structure, thereby enabling a more complete characterization of loop semantics than any single representation alone. The proposed framework decomposes loop invariant inference into two phases: template selection and template instantiation. In the first phase, a structure-aware convolutional neural network(CNN) predicts the most suitable invariant template category directly from program structure. The model extracts syntactic features from Abstract Syntax Tree (AST) embeddings using Word2Vec, and semantic graph features from Control Flow Graph (CFG) and Data Dependence Graph (DDG) representations using Node2Vec. These representations are integrated through a gated fusion layer to capture complementary structural information and determine the appropriate invariant template category for a given loop. In the second phase, the selected template is instantiated using execution traces to infer its parameter values, yielding a concrete invariant candidate that is subsequently validated using a Satisfiability Modulo Theories (SMT) solver.

Experimental results on a diverse set of benchmark programs demonstrate that the proposed approach effectively supports fully automated loop invariant inference by accurately predicting appropriate invariant template directly from loop code. The effectiveness of the framework stems from its ability to perform deterministic and targeted template selection through a comprehensive structural analysis of the loop. By jointly leveraging complementary information from AST, DDG, and CFG, the model constructs a unified representation that captures both the syntactic structure and semantic dependencies of the program. As a result, the approach avoids the computational overhead associated with exploring and verifying redundant templates, replacing heuristic guess-and-check strategies with predictable, representation-driven synthesis. Guided by the adaptive gating mechanism, the model consistently identifies appropriate templates with high precision across loops with varying arithmetic and control-flow complexity. Consequently, the framework achieves high automation and computational efficiency while successfully synthesizing valid invariants for complex loop behaviors.

The main contributions of this article are summarized as follows:

- We propose a structure-aware loop invariant inference framework that jointly analyzes AST, CFG, and DDG representations to automatically predict the most suitable invariant template from six predefined classes through a learning-based template selection mechanism.
- We introduce a two-phase inference pipeline that combines CNN-based template selection, execution-driven coefficient instantiation, and SMT-based formal verification. The framework reduces the invariant search space while guaranteeing soundness through exhaustive SMT validation.
- We construct a benchmark suite of 100 annotated C loop programs covering six invariant template classes, including both established benchmark programs and newly synthesized examples to improve class balance and evaluation diversity.

The rest of this paper is structured as follows. Section 2 examines related studies. Section 3 presents the essential information about the approach. Section 4 explains the proposed approach for both template generation and invariant inference. The experimental setup is described in detail in Section 5. Section 6 analyzes and discusses the results of the experiments. Section 7 presents threats to validity. Finally, the study is summarized in Section 8.

## 2 Related work

This section discusses previous researches on loop invariant inference, dividing it into traditional and learning-based approaches.

**Traditional methods**: encompass various techniques such as constraint solving [22,23], abstract interpretation frameworks [24–26], logical abduction processes [27,28], and model checking techniques [29,30]. Other notable strategies include Craig interpolation [31,32], syntax-guided synthesis methods [33,34], and approaches based on counterexample-guided abstraction refinement [35,36]. Additionally, recurrence analysis [37–39] has been explored. For example, Eldarica [29] utilizes a Horn clause solver rooted in model checking, while SMT solvers like CVC4 and CVC5 [33,34] incorporate syntax-guided synthesis to generate invariants. Although these traditional approaches perform reliably in many settings, they struggle with nonlinear program behaviors and large search spaces, limiting their practicality in real-world software analysis. As a result, they are generally less scalable and less efficient than modern learning-based techniques.

**Learning-based methods**: A trend has emerged in which the guess-and-check paradigm dominates, as seen in works such as [12–14,16,29]. These methods involve iteratively proposing candidate invariants and verifying their correctness, although they often lack formal completeness guarantees. Despite that, they tend to outperform earlier approaches in practice. Many of these methods leverage machine learning models, including decision trees [20], reinforcement learning [16,21], and continuous logic networks [13,40], to generate promising invariant candidates.

Compared to prior learning-based approaches, including decision trees, ICE-style algorithms, and even recent LLM-based methods [41], our framework offers a more targeted and scalable solution to invariant inference. Rather than generating invariants directly, a process that often struggles with complex logical structures, depends on manually crafted templates, or requires extensive prompting and fine-tuning, our method decomposes the task into two phases: generating an invariant template using features extracted from rich program representations (AST, CFG, and DDG), and then solving this template using concrete variable values. This design reduces search space, increases the level of automation, and enables the model to capture more intricate logical relationships, generating invariants more accurately and efficiently compared to other learning-based approaches.

## 3 Background

In this section, we begin by introducing the loop invariant inference problem and then provide the necessary background on source code representations.

### 3.1 Loop invariant

The goal of loop invariant inference is to determine an invariant that can be used to show that a program satisfies its desired correctness properties formally. For a loop of the form **while** *C* **do** *ST*, the classical Hoare logic rule states that the invariant must satisfy


$$\begin{array}{c} \frac{Pre\;\Rightarrow \;Inv\;\;\{Inv\wedge C\}\;ST\;\{Inv\}\;\;(Inv\wedge \neg C)\;\Rightarrow \;Post}{\left\{Pre\}\;\text{while }C\;\text{do }ST\;\{Post\right\}} \end{array}$$

The rule above corresponds to the classical Hoare logic [4] formulation for establishing the partial correctness of a loop. The *Pre* denotes the *precondition*, specifying what must hold before the loop begins. The *Inv* represents the *loop invariant*, a logical assertion that remains true before and after each iteration. The *C* refers to the *loop condition* (or guard) that determines whether the loop continues executing. The *ST* denotes the *loop body*, which is the sequence of statements executed in each iteration. Finally, *Post* represents the *postcondition*, describing the property that must hold once the loop terminates. To prove the correctness of the Hoare triple {*Pre*} **while** *C* **do** *ST* {*Post*}, Three conditions must be satisfied:

1. **Initialization:** Pre⇒Inv, meaning the invariant holds before the first iteration.
2. **Preservation:** {Inv∧C} ST {Inv}, meaning if the invariant and the loop guard hold, executing the loop body re-establishes the invariant.
3. **Termination:** (Inv∧¬C)⇒Post, meaning when the loop terminates, the invariant and the negation of the guard imply the postcondition.

### 3.2 Source code representations

Source code can be represented in various forms, with textual and graph-based representations being the most common [42]. Textual representations consist of the source code itself, written in a programming language, and serve as the standard format for software development and analysis. Among these, AST is used as an intermediate representation for syntactic analysis and code understanding. Graph-based representations, in contrast, provide abstract models of the code to support program comprehension, optimization, and static analysis. Notable examples include CFG, which depicts the sequence of execution paths, and Data Dependence Graphs DDG, which illustrate dependencies between data elements in a program. These graphical representations are not literal visualizations of the code, but serve as essential tools for automated analysis and program reasoning.

#### 3.2.1 Abstract syntax trees.

AST is a tree-like intermediate representation of source code that captures its syntactic structure. Each node in an AST corresponds to a language construct, such as expressions, statements, or control structures. AST is widely used in compilers, interpreters, and static analysis tools to facilitate tasks such as syntax checking, code optimization, program transformation, and understanding the logical flow of a program. By abstracting away low-level syntax details, AST provides a structured way to analyze and manipulate code programmatically. For example, Fig 1b shows the AST generated from the C code in Fig 1a. In this representation, the loop structure and assignments are decomposed into a hierarchy of syntactic nodes, allowing the parser to clearly capture constructs such as the while statement, the internal if statement, and the update expressions for *a*, *b*, *i* and *n*. Although the AST provides a precise syntactic breakdown of the loop and exposes how each statement is nested within the control structure, it remains limited for deeper program analysis. As a result, AST alone cannot reveal essential semantic properties, such as loop-carried dependencies or the effect of conditional execution, which are necessary for understanding the computational behavior of the program.

**Fig 1:**
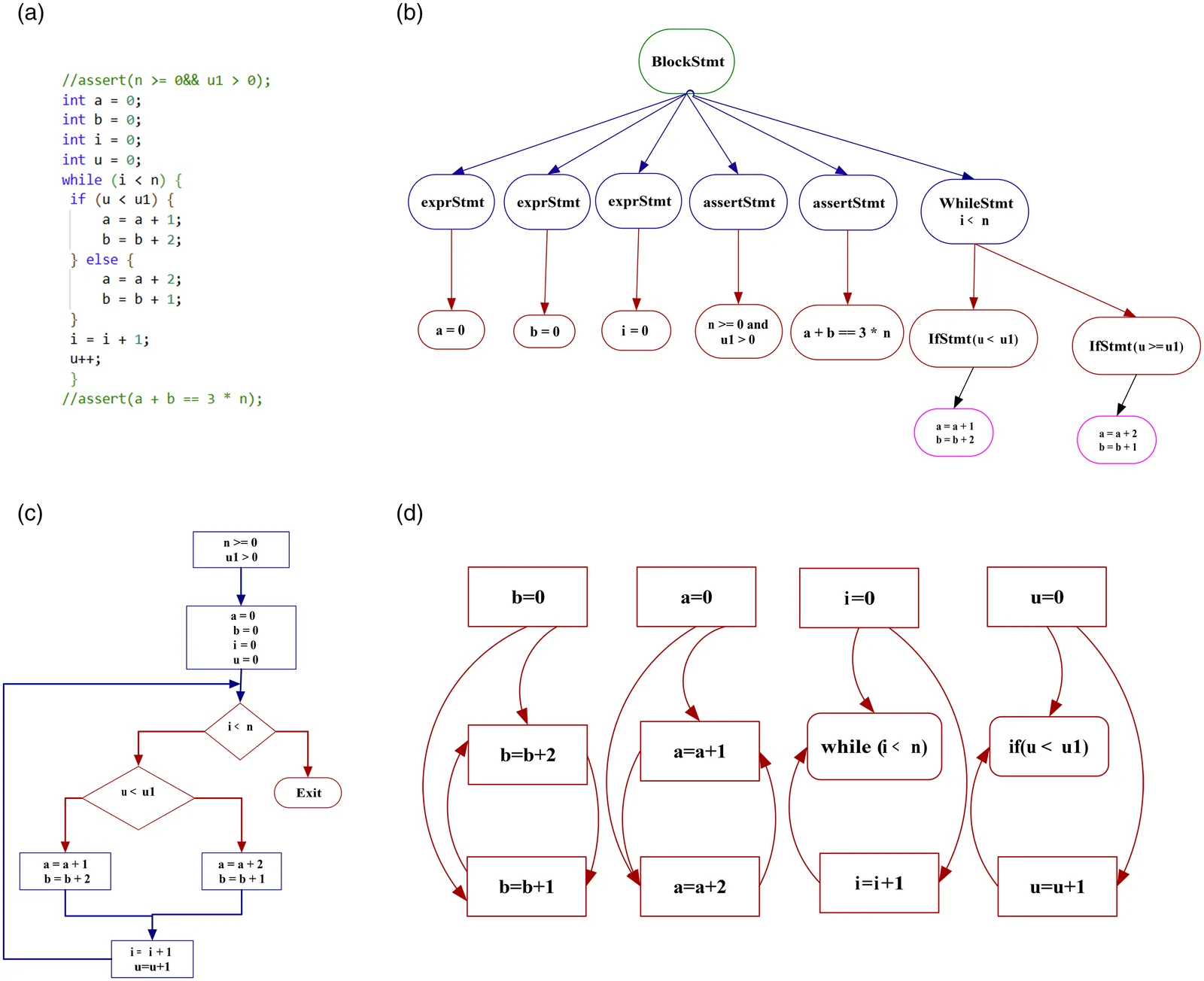
Different representations of source code. (a) C code example (b) AST (c) CFG (d) DDG.

#### 3.2.2 Control flow graph.

CFG is a graphical representation of a program that illustrates the order in which individual statements or instructions are executed. In a CFG, nodes represent basic blocks of code, and edges indicate the possible flow of control between these blocks. CFG is widely used in compiler design, program analysis, and optimization, as it helps identify execution paths, detect unreachable code, and support techniques such as loop analysis. By abstracting the flow of a program, the CFG enables a clear understanding of its runtime behavior. For example, Fig 1c illustrates the CFG of the C program in Fig 1a. Every time the loop is executed, the code considers the control input *i*, *u* and updates the values of *a* and *b* according to this control. This execution is indicated by the node representing *u* > *u*1, from which two potential paths start. If *u* > *u*1, the edge leads to *a* = *a*+1 and *b* = *b*+2, else leads to *a* = *a*+2 and *b* = *b*+1 as shown in the corresponding node. Then the shift moves to *i* = *i*+1 and *u* = *u*+1. Together, these paths define how the program iteratively computes the final value of *a* and *b* based on the input *u* and the initial value *u*1, eventually reaching the exit condition.

#### 3.2.3 Data dependence graphs.

DDG represent how data values in a program depend on each other. Nodes correspond to operations or statements, and edges indicate the flow of data between them. DDGs are particularly useful for detecting dependencies, enabling safe parallel execution, and optimizing instruction ordering. The Fig 1d illustrates the flow of data values between statements of the given code in Fig 1a, rather than the control flow. In this DDG, nodes represent individual statements that produce or consume variables, while edges indicate data dependencies, showing how the output of one statement is used as input by another. For example, the assignment *a* = 1 feeds into the computation *a* = *a*+1, and the assignment *a* = *a*+2 depends on the current value of *a*. This representation makes it clear which statements must be executed in a particular order to respect the data dependencies, which is critical for optimizations, parallelization, and understanding the propagation of values through the program.

## 4 Proposed approach

In this section, we present an overview of our approach, which consists of two main stages: (1) invariant template selection and construction, and (2) invariant instantiation and validation, as illustrated in Fig 2.

**Fig 2:**
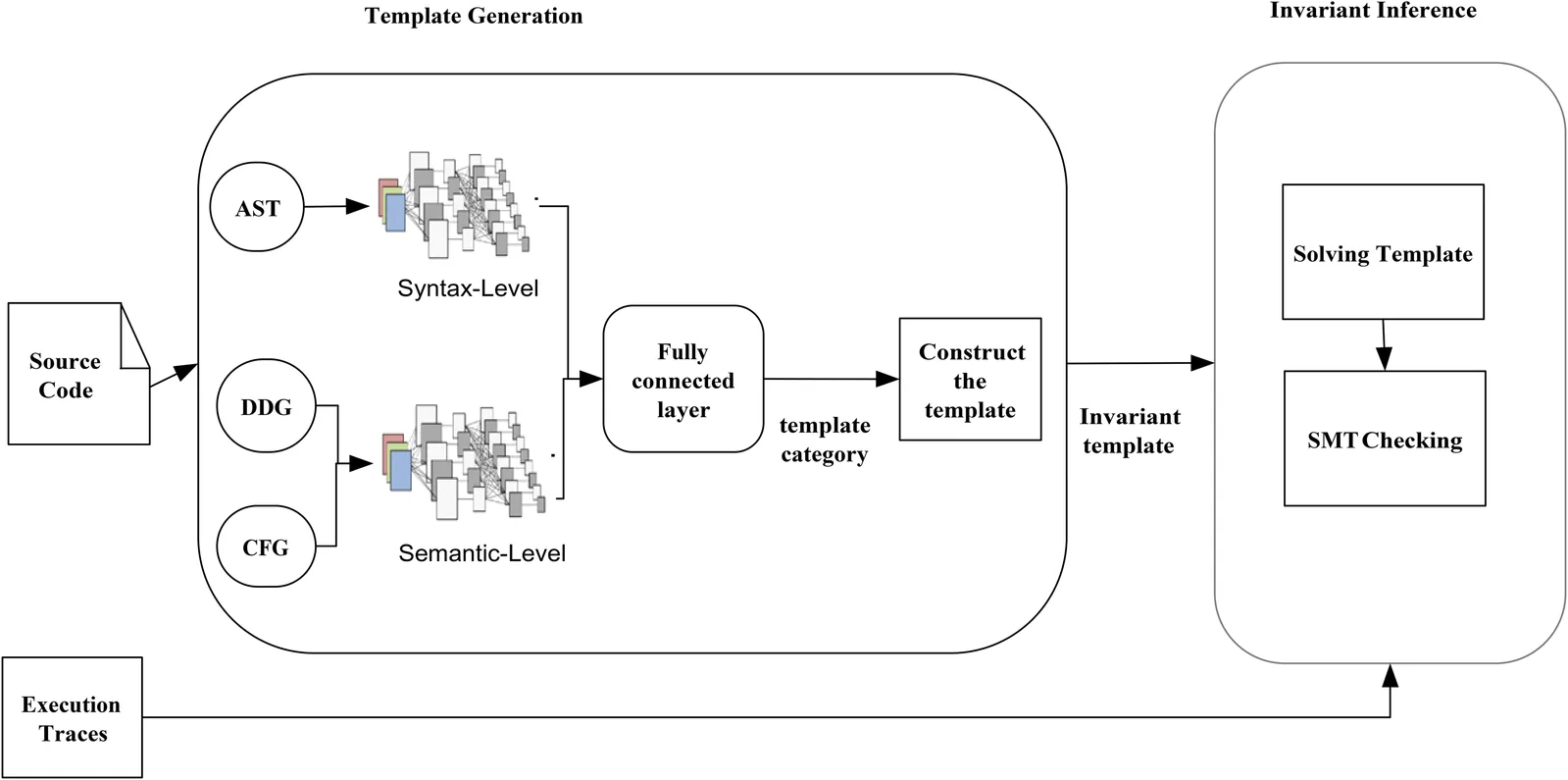
Overview of the proposed approach. The approach consists of two main stages: (1) invariant template selection and construction, and (2) invariant instantiation and validation.

### 4.1 Template selection and construction

The template construction phase follows a three-step process comprising source code parsing, transformation of program representations into vector embeddings, and application of the model. Together, these steps enable the identification of an appropriate template category. The following subsections provide a detailed description of each step.

#### 4.1.1 Loop parsing.

**Abstract Syntax Tree**: In this stage, we construct a unified representation by parsing the program’s source code into its AST. The following AST nodes are parsed, which together capture the essential structural elements of the loop:

- Nodes of the loop statement and Condition.
- Nodes of *Pre* and *Post* assertion
- Nodes of variable declaration.
- Nodes of variable update statements.

In our experiments, we parse C source code into AST using the Python library pycparser, which is widely used for parsing and analyzing C programs. The AST generated by pycparser enable programmatic analysis and manipulation of C code, making it a valuable tool for tasks such as program verification, static analysis, and code transformation.

**Algorithm 1: Extract Ast Nodes From Loop Source Code**



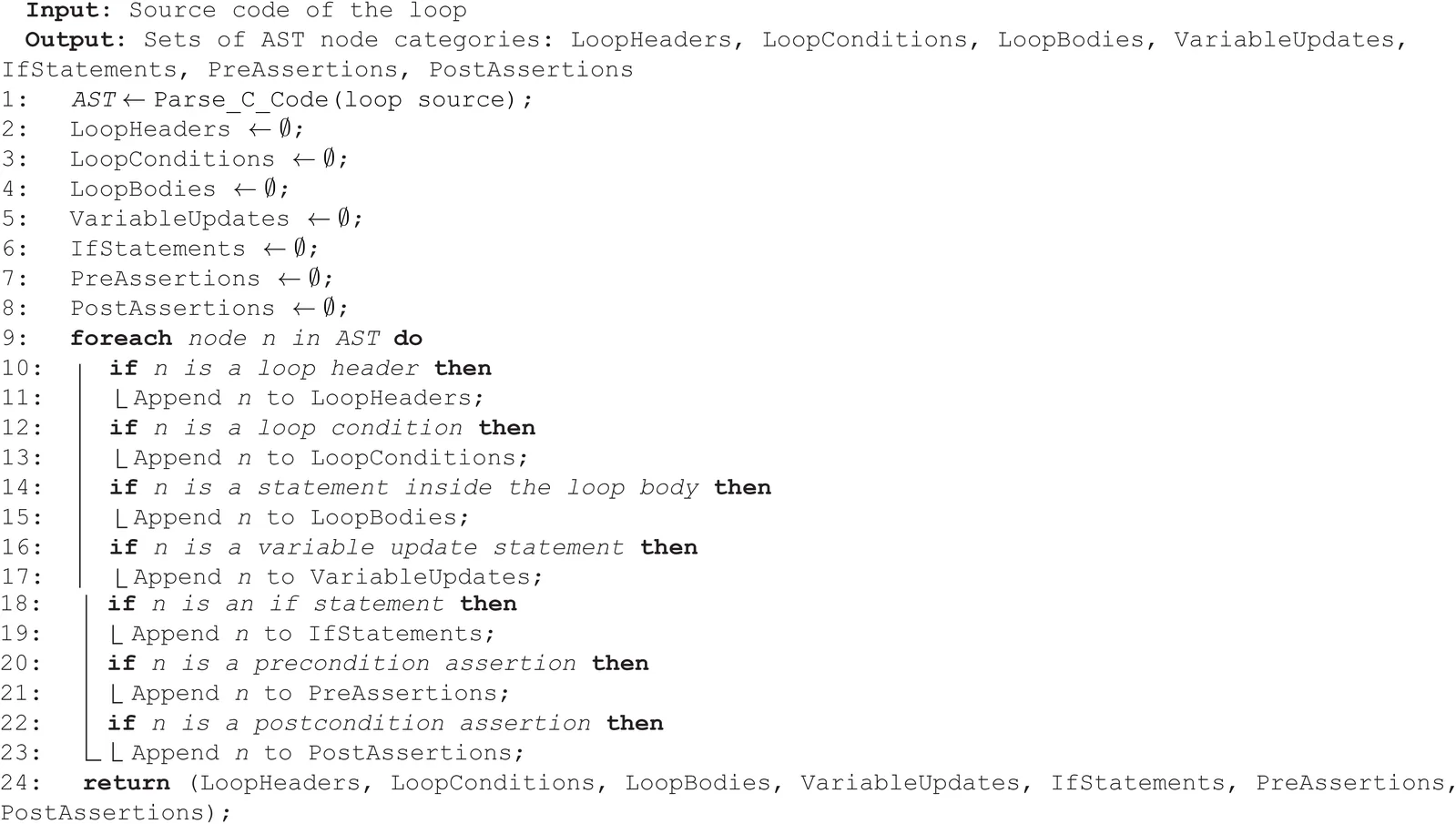



The process of parsing the AST from C source code using pycparse is described in Algorithm 1. The algorithm takes the loop source code as input, converts it into its corresponding AST representation, and systematically traverses the resulting tree to extract structural components of the loop, including headers, conditions, body statements, variable-update statements pre and post assertions, and embedded conditional constructs. The AST is traversed node by node, and each node is classified according to its syntactic role within the loop. The loop header constructs and their associated condition expressions are identified explicitly, while all statements enclosed within the loop’s body are collected to preserve the structural sequence of the loop. Additionally, the algorithm isolates variable update statements, such as increments, decrements, and self-referential assignments, as well as embedded if statements that influence control flow within the loop. Finally, the algorithm returns the categorized sets.

**Control Flow Graph**: To enable deeper semantic understanding of loops during invariant inference, our approach combines precise structural graphs (CFG and DDG) with contextual syntactic information extracted from the AST. This section describes the procedure for constructing the CFG of each loop, while the following section details the generation of the DDG. For CFG construction, we build a graph $G=(N,E,n_{0},n_{e})$ for every loop in our dataset, where *N* is the set of nodes, *E* is the set of directed edges, *n*_0_ is the loop header, and $n_{e}$ is the loop exit. Each node n∈N represents a basic block inside or related to the loop structure, including loop entry, loop body, branch blocks, and loop exit blocks. Each edge $e=(n_{i},n_{j})\in E$ denotes a possible control transfer from block $n_{i}$ to block $n_{j}$, capturing behaviors such as branching conditions and back edges that characterize loop iteration. Algorithm 2 constructs the Control Flow Graph (CFG) directly from loop source code. The input program is first decomposed into a sequence of statements, each mapped to a corresponding CFG node. Edges are then introduced to reflect the sequential execution order, while control constructs, such as loop headers, conditional branches, break, and continue statements, are identified through pattern matching. A stack-based track is employed to correctly handle nested control structures, enabling proper construction of back edges and exit transitions. The resulting graph includes both loop iteration and exit edges, yielding a compact source-level CFG representation.

**Algorithm 2: Constructing a CFG from Loop Source Code**



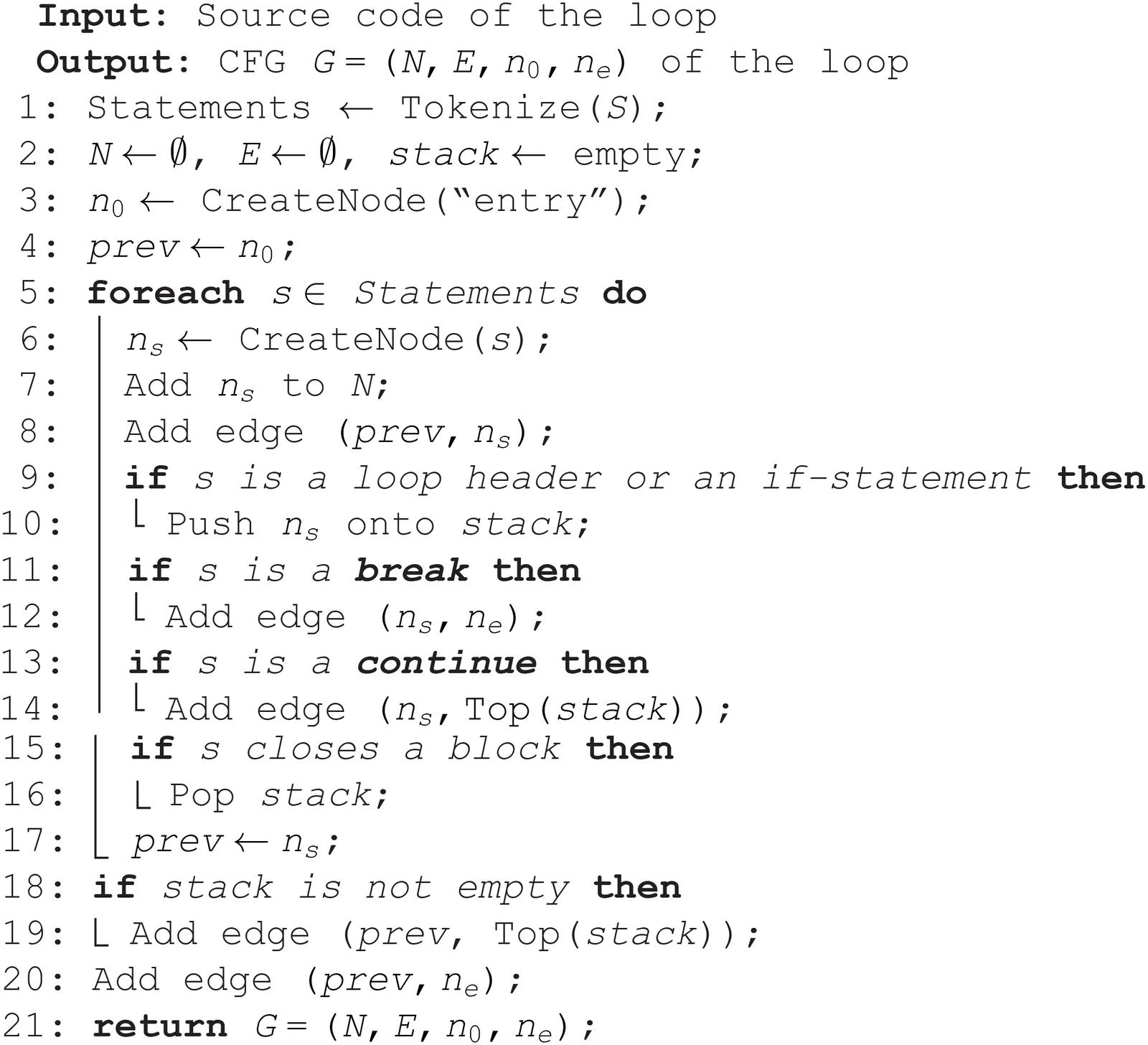



We construct a custom CFG to avoid low-level compiler artifacts and improve robustness. It removes the noise that can distort graph embeddings and instead preserves only high-level semantic structure relevant for invariant learning. It also enables analysis of loop snippets or isolated program fragments without requiring full compiler environments or resolved dependencies.

**Data Dependence Graph**: We construct the DDG from the loop source code using use-definition analysis [43]. For each statement *n*, two sets are extracted: the *defined* set *Def*[*n*], containing variables assigned or modified at *n*, and the *used* set *Use*[*n*], containing variables whose values are read at *n*. A directed edge (*m*, *n*) is introduced if a variable v∈Def[m] appears in *Use*[*n*], representing a data dependency between the two statements. Each DDG node is further annotated with its corresponding Control Flow Graph (CFG) node to preserve control-flow context. This construction captures variable interaction patterns within the loop, providing structural information essential for identifying the mathematical form of the invariant. Algorithm 3 formalizes DDG construction by computing use-definition information for each statement and deriving data dependencies as directed edges between statements.

**Algorithm 3: Constructing DDG from Loop Source Code**



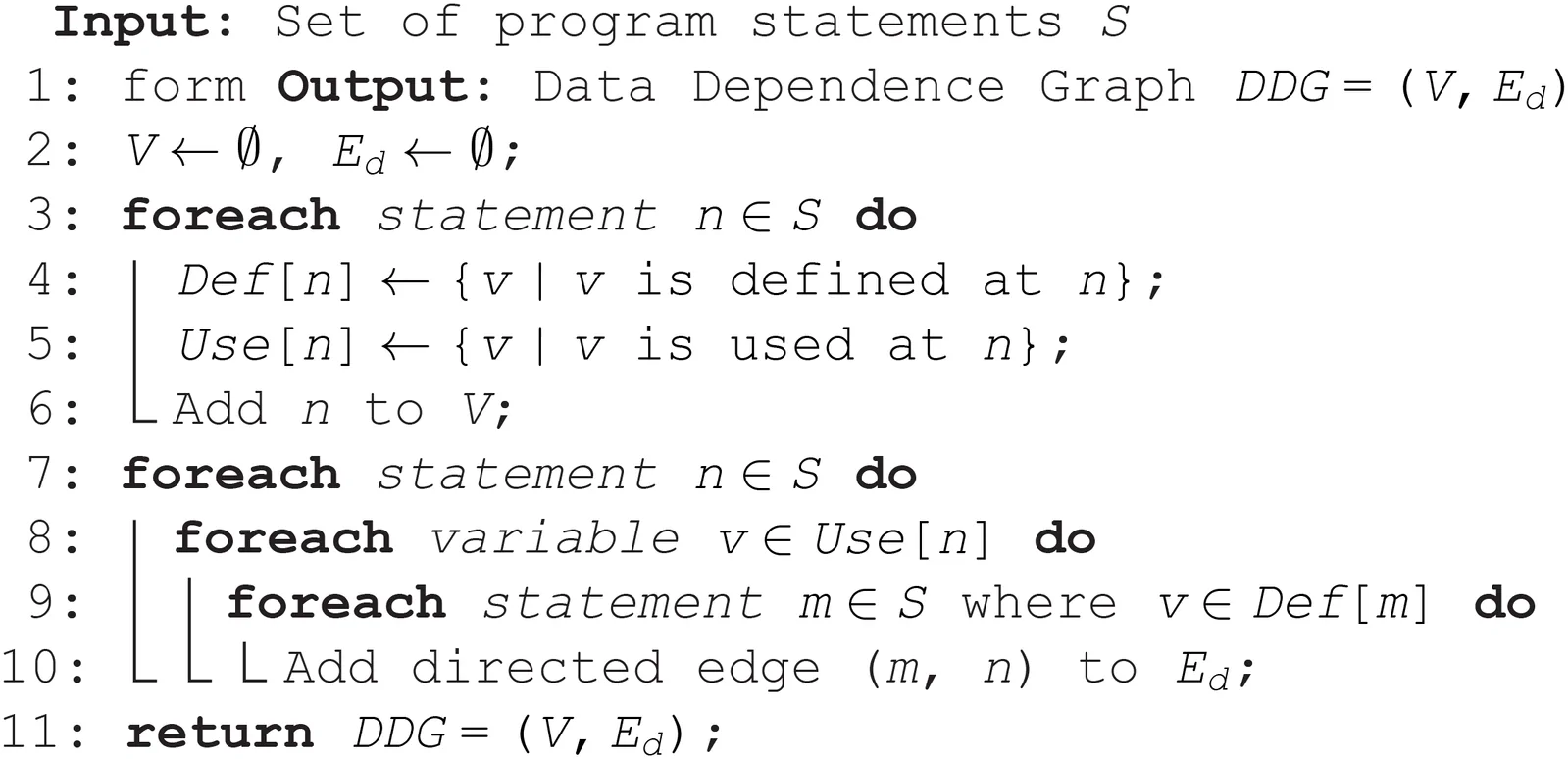



#### 4.1.2 Vector embeddings.

After extracting the syntactic features (AST) and semantic graph features (CFG and DDG) from the loop structures, these features are then mapped into fixed-size numerical vectors suitable for learning model. The goal of this mapping is to transform the high-level structural and semantic information into representations that can be effectively processed by the neural network model, thereby preserving critical contextual and dependency information. We utilize Word2vec [44] to embed AST nodes into continuous vectors, capturing the semantic relationships among syntax elements. For semantic graphs, Node2vec [45] is employed to generate embeddings for CFG and DDG nodes, encoding the control flow and data dependencies into low-dimensional vectors. These embedded vectors serve as input features to our neural network model, which select the category of the invariant template.

**Word2vec**: In our approach, we employ Word2Vec to transform the syntactic structure of loops into meaningful vector embeddings. Instead of using Word2Vec for natural language tokens, we treat AST nodes and their hierarchical relations as the vocabulary and contextual sequences on which the embedding model is trained. This allows the model to learn distributed representations that encode syntactic roles, structural relations, and recurring patterns found in loop constructs. By embedding AST nodes into a continuous vector space, we obtain a syntactic representation that supports more effective reasoning about loop behavior. These embeddings enhance the invariant inference process by enabling the model to identify structural cues and patterns correlated with invariant properties, thereby improving accuracy and capturing deeper syntactic insights from the loop’s code structure.

**Node2Vec** is employed to transform the structural characteristics of both CFG and DDG into meaningful low-dimensional vector embeddings. By performing biased random walks over these graphs, Node2Vec captures both local connectivity patterns and higher-order structural relationships among nodes. This process allows the embedding model to learn representations that encode control dependencies, data interactions, and structural roles of statements within the loop. Mapping the CFG and DDG into a continuous vector space enables downstream learning components to effectively leverage graph-based semantics, ultimately improving the accuracy and robustness of invariant inference.

#### 4.1.3 Template selection model.

The proposed model integrates syntactic information derived from the AST with semantic information encoded in the CFG and DDG to select the most suitable invariant template category for a given loop program. The model adopts a dual-level architecture inspired by hierarchical representation learning, where the syntactic and semantic-graph representations are processed independently through. The outputs of the two levels are subsequently combined through a gated merging layer that learns the optimal contribution of each representation to the template selection decision. Fig 3 illustrates the overall model architecture, which consists of two levels: a syntax level that processes AST embeddings and a semantic-graph level that processes CFG and DDG embeddings, followed by a gated merging layer and a classification layer that outputs the predicted invariant template category.

**Fig 3:**
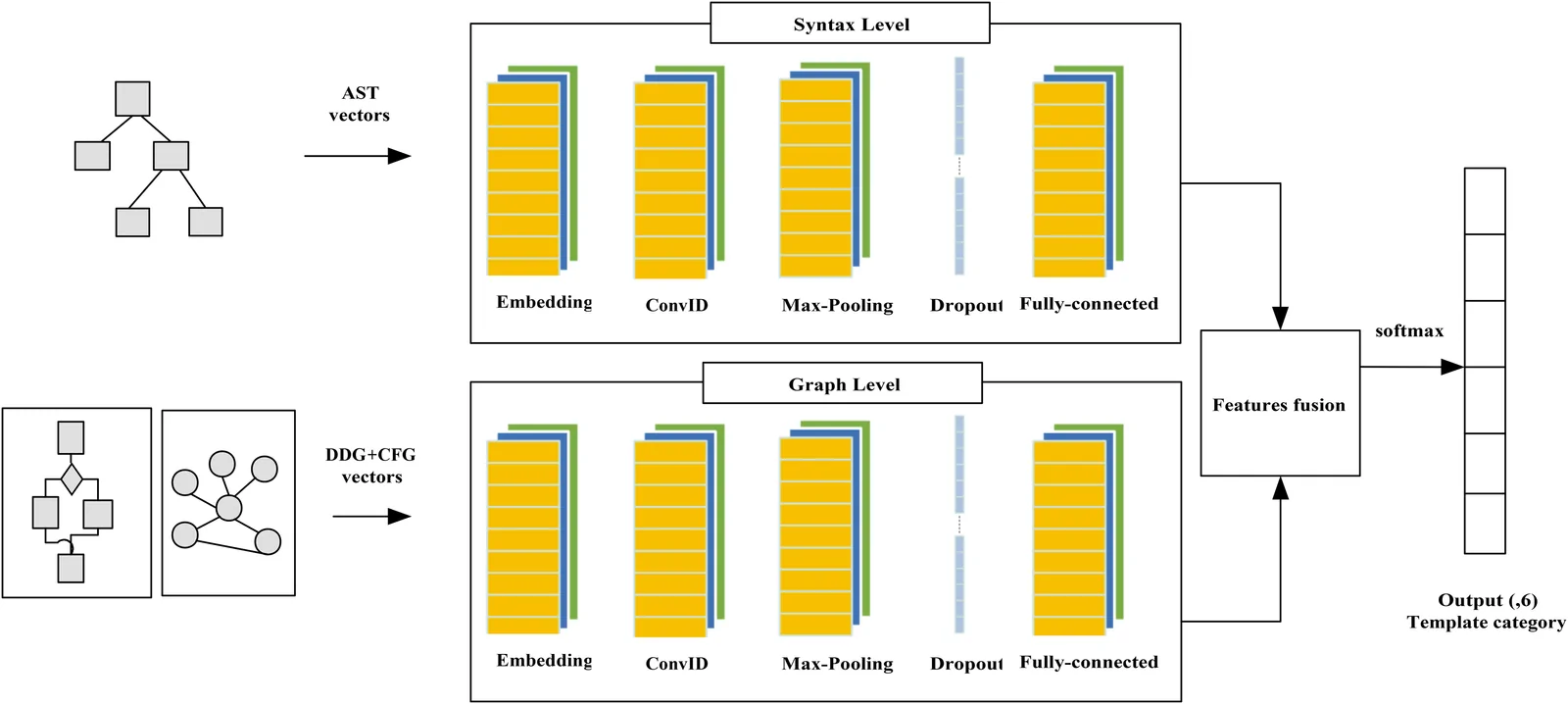
Overview of the template construction model. The model combines AST embeddings at the syntax level and CFG/DDG embeddings at the semantic-graph level, followed by gated merging and classification of the invariant template category.

**Dual-Level Feature Extraction:** As described in Fig 3, the model processes program representations through two levels corresponding to syntactic and semantic levels. At the syntactic level, the AST embedding is passed through a one-dimensional convolutional layer to capture local patterns, followed by global max pooling, dropout, and a fully connected layer to produce a fixed-size feature vector. At the semantic-graph level, CFG and DDG embeddings are concatenated to form a unified representation that captures complementary control-flow and data-dependence information. This representation is processed using the same convolutional architecture, yielding a semantic feature vector that encodes structural properties relevant to invariant template selection.

**Gated Merging Layer.** The outputs of the two levels are combined through a gated merging layer that learns the relative contribution of each representation level to the template selection task. Rather than simple concatenation, the gating mechanism adaptively weights the syntactic and semantic-graph representations based on the structural characteristics of the input loop. Formally, the merged representation $G_{m}$ is computed as:


$$\begin{array}{c} G_{m}=integrate(\phi (U_{f}\cdot X_{s}+d_{s}),\phi (U_{g}\cdot X_{g}+d_{g})) \end{array}$$ (1)

where $X_{s}$ and $X_{g}$ denote the feature vectors derived from the AST and the combined CFG–DDG branches, respectively. The matrices $U_{f}$ and $U_{g}$ and bias terms $d_{s}$ and $d_{g}$ are learnable parameters, and ϕ(·) denotes the ReLU activation function. The operator integrate(·,·) represents a learnable gated fusion mechanism that combines the two representations into a unified feature vector. This design enables the model to adaptively emphasize the most informative representation for each input, resulting in more effective template selection compared to fixed combination strategies.

**Example**: Consider the loop in Fig 1a and its corresponding AST (Fig 1b), CFG (Fig 1c), and DDG (Fig 1d). The combined representations provide rich information for learning invariant templates. From the AST, the exprStmt nodes show that all loop updates are linear integer increments, such as *a* = *a* + 1, *b* = *b* + 2, *a* = *a* + 2, *b* = *b* + 1, *i* = *i* + 1, and *u* = *u* + 1. Moreover, the postcondition assert(a + b==3*n) further indicates a linear relation among program variables, suggesting that the loop invariant also has a linear form. From the CFG, both branches of the conditional update *a* and *b* before merging at the loop back-edge. This shows that *a* and *b* are consistently modified together in every iteration, suggesting a unified linear relationship rather than a disjunctive invariant. From the DDG, the use-definition chains indicate that *a*, *b*, and *i* evolve through additive dependencies across iterations, with each value derived linearly from the previous iteration. The absence of nonlinear dependencies further supports a linear invariant form. By combining syntactic evidence of linear updates from the AST, consistent branch-wise updates from the CFG, and linear dependency from the DDG, the dual-level architecture with gated fusion identifies the invariant template category as equality_linear.

#### 4.1.4 Template category selection and training.

The merged representation ${𝐆}_{m}$ is passed to a classification layer that selects the most likely invariant template category from a predefined category space: $𝒯=\{t_{1},t_{2},...,t_{6}\}$ where each $t_{i}$ corresponds to one of six invariant template categories: equality_linear, equality_nonlinear, inequality_linear, inequality_nonlinear, conjunctive, and disjunctive. The predefined category space *T* represents a minimal set of structural distinctions that remain largely independent of specific program domains. While this design introduces a limited manual specification, it eliminates the need for manually crafting invariant templates for individual programs. During training, the model learns to associate structural and semantic characteristics of loops with appropriate template categories by minimizing a cross-entropy loss. At inference time, the selected category $t_{i}\in 𝒯$ determines the structural form of the invariant, which is subsequently instantiated over the variables appearing in the loop to construct the invariant template. The probability distribution over these categories is computed as:


$$\begin{array}{c} y=softmax\left(UG_{m}+d\right), \end{array}$$ (2)

where *U* denotes the weight matrix of the classification layer and *d* is the corresponding bias vector. The entire model is trained end-to-end using the Adam optimizer [46] with a cross-entropy loss function.

#### 4.1.5 Template construction.

Following template category selection, the framework identifies the corresponding parameterized invariant template. Each of the six predefined categories defines a distinct structural form over the program variables, with unknown coefficients to be inferred. Specifically, the selected category determines whether the invariant is expressed as a linear equality, a nonlinear equality, a linear inequality, a nonlinear inequality, a conjunctive, or a disjunctive form. The resulting symbolic template defines the search space for concrete invariants and is subsequently instantiated using execution traces to recover coefficient values. The obtained candidate is then formally validated using an SMT solver.

**Example**: Following the selection of the equality_linear category for the example in Fig 1a, the framework constructs a parameterized invariant template consistent with the predicted class. Variables are included in the template according to two criteria: (1) they are actively updated within the loop body, and (2) they appear in the postcondition or are directly data-dependent on postcondition variables through the loop’s dependence structure. For the Fig 1a example, *a*, *b*, and *i* satisfy both conditions. They are updated in every loop iteration and are directly related to the postcondition a + b==3*n through the loop counter *i*. Based on these observations, the framework constructs the following parameterized template with unknown coefficients *c*_1_, *c*_2_, and *c*_3_:


$$\begin{array}{c} c_{1}\cdot a+c_{2}\cdot b+c_{3}\cdot i=0 \end{array}$$ (3)

This parameterized form defines the search space for the concrete invariant and serves as input to the subsequent constraint instantiation stage, where execution traces are used to infer the values of the unknown coefficients.

### 4.2 Loop invariant inference

In the second phase of the proposed framework, the constructed template is instantiated by solving for the unknown coefficients that satisfy the loop semantics. Therefore, execution traces are collected by running the loop over a diverse set of input values, recording the values of program variables at each iteration. These observations are used to derive constraints on the template parameters. The resulting solving procedure varies across template categories, reflecting the underlying mathematical structure of the induced constraint systems.

#### 4.2.1 Polynomial template solving.

We construct templates of polynomials over loop variables to represent equality or inequality constraints. In a nonlinear polynomial, the template contains a set of *terms* that encode nonlinear combinations of the variables up to a specified degree. For example, given the variables {*x*, *y*, *z*} and degree 2, the generated template contains the following set of 10 monomial terms: $\{1,x,y,z,xy,xz,yz,x^{2},y^{2},z^{2}\}.$ More generally, for *n* variables and degree *d*, we enumerate all monomials of degree up to *d*, resulting in a set of (|V|+dd) terms. The polynomial invariants over these terms are inferred by solving the corresponding equations using concrete values. Specifically, we construct a polynomial equation template of the form:


$$\begin{array}{c} c_{1}\tau_{1}+c_{2}\tau_{2}+...+c_{n}\tau_{n}\;\sim \;0, \end{array}$$ (4)

Where each $\tau_{i}$ represents a monomial term over the loop variables, and ~∈{=,≤,≥} indicates the type of constraint.

**Algorithm 4: solvePolyTemplate**



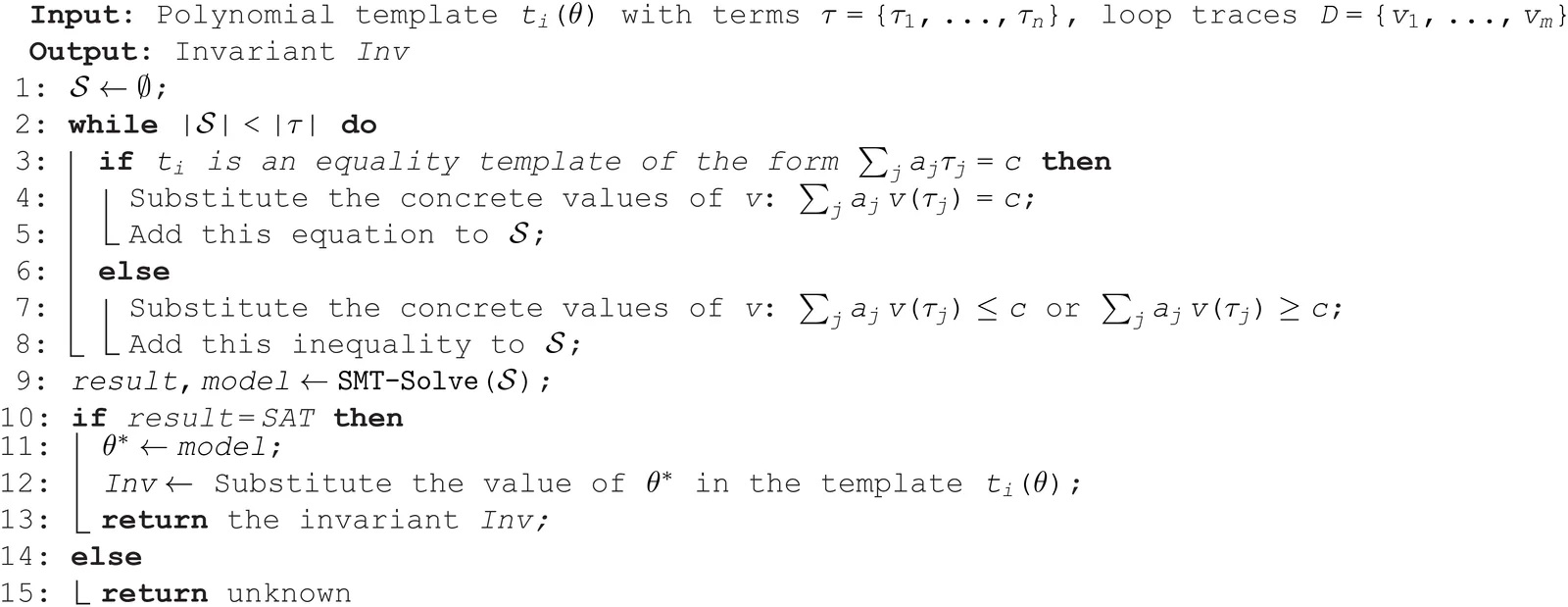



Algorithm 4, solvePolyTemplate, infers the coefficients of a polynomial template $t_{i}(\theta )$ using a set of concrete loop traces $D=\{v^{\left(1\right)},...,v^{\left(m\right)}\}$. The template $t_{i}(\theta )$ is represented as a linear combination of terms $\tau =\{\tau_{1},...,\tau_{n}\}$, where each $\tau_{j}$ may be a linear or nonlinear monomial over the loop variables. The algorithm maintains a set of constraints 𝒮, initially empty. It iteratively substitutes concrete variable values from the collected loop traces into the template terms until the number of generated constraints matches the number of unknown template coefficients(|𝒮|=|τ|). All generated constraints are accumulated into 𝒮. Once sufficient constraints have been collected, the solver is invoked through SMT-Solve(𝒮) to compute a coefficient assignment $\theta^{*}$ satisfying all constraints simultaneously. If the constraint system is satisfiable, the resulting model provides concrete values for the unknown template parameters, which are substituted into the template $t_{i}(\theta )$ to instantiate the candidate invariant $Inv=t_{i}(\theta^{*})$. If the constraint system is unsatisfiable, no coefficient assignment exists for the current template $t_{i}(\theta )$, the solver returns unknown.

#### 4.2.2 Disjunctive template solving.

Disjunctive invariants arise when a loop can follow different patterns of execution that a single invariant cannot adequately describe. While an ordinary invariant summarizes one common relation satisfied by all reachable states, a disjunctive invariant captures the behavior where the loop may progress in several distinct states depending on the branching conditions.

The loop shown below involves two variables, *x* and *y*. Because the loop contains multiple control-flow paths, our analysis of the abstract AST, CFG, and DDG construct that an appropriate disjunctive invariant template for this loop is x<a∨x+y<b.

assert(n > 0);

int x = 0;

int y = 0;

while (x + y < 2*n) {

if (x < n)

x++;

else

y++;

}

assert(x + y>= 2*n);

**Algorithm 5: Disjunctive Template Solving**



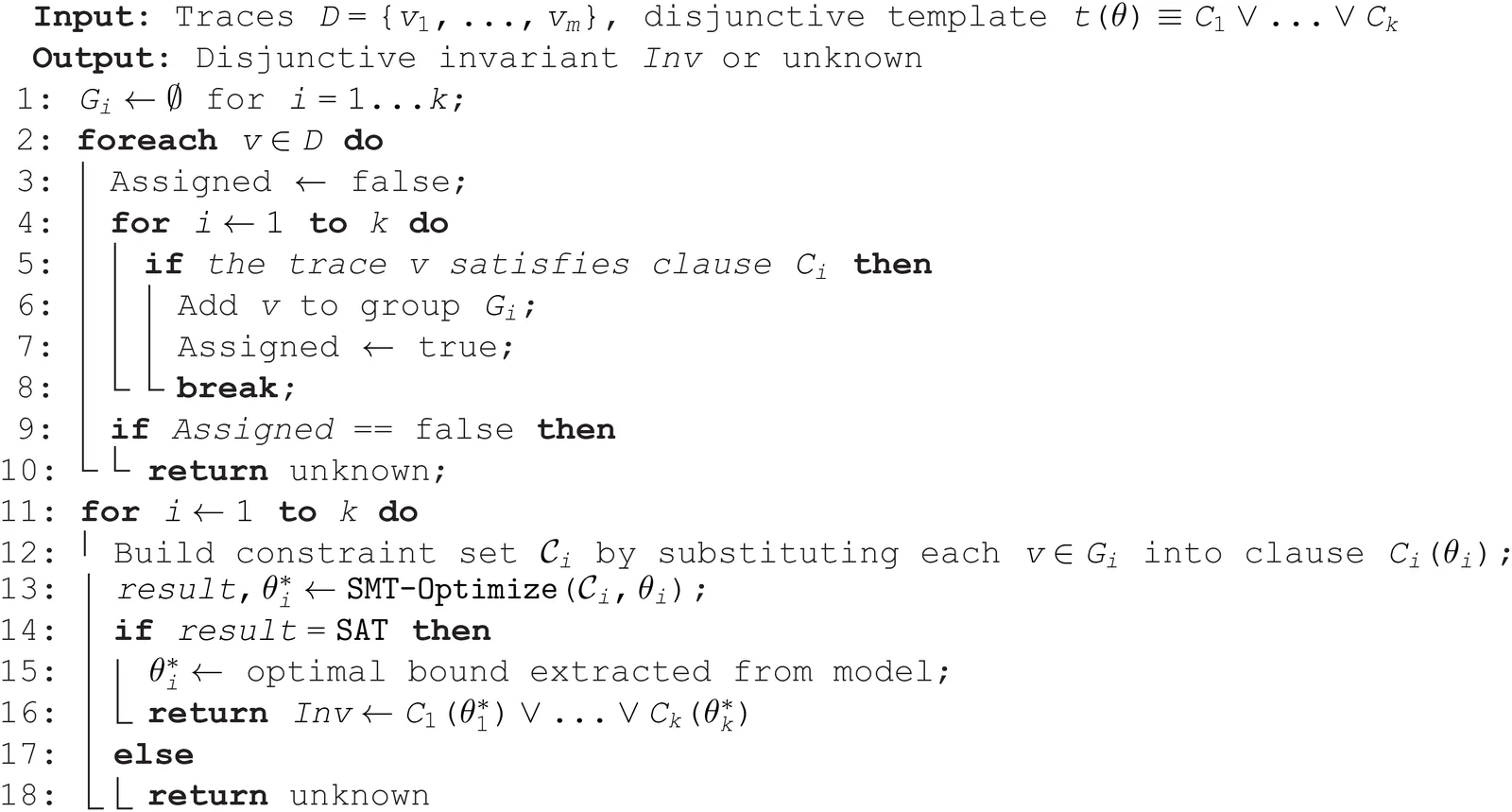



Algorithm 5 presents a general procedure for solving disjunctive invariants from a set of loop traces. The input consists of the set of traces $D=\{v_{1},...,v_{m}\}$ and a disjunctive template $t(\theta )\equiv C_{1}\vee ...\vee C_{k}$, where each $C_{i}$ is a clause that contains some parameters to be solved. The algorithm proceeds in two main stages. First, it checks whether the trace can satisfy any clause $C_{i}$ for some value of the unknown bound. If a trace cannot satisfy any clause, the algorithm returns unknown, indicating that no invariant exists. Otherwise, traces are grouped into sets $G_{i}$ according to the clause they satisfy. Second, for each clause $C_{i}$, the algorithm builds a constraint set ${𝒞}_{i}$ by substituting the concrete trace values of each $v\in G_{i}$ into the clause $C_{i}(\theta_{i})$. An SMT-based optimization procedure ($\text{SMT-Optimize}({𝒞}_{i},\theta_{i})$) is then invoked to compute the maximum bound $\theta_{i}^{*}$ that ensures all traces in $G_{i}$ satisfy the clause $C_{i}$. If $\text{SMT-Optimize}({𝒞}_{i},\theta_{i})=SAT$, the optimal bound $\theta_{i}^{*}$ is extracted from the satisfying model, and the algorithm constructs the disjunctive invariant by combining all clauses with their computed optimal bounds: $Inv=C_{1}(\theta_{1}^{*})\vee C_{2}(\theta_{2}^{*})\vee ...\vee C_{k}(\theta_{k}^{*})$. If the optimization returns UNSAT, meaning no valid maximum bound exists for clause $C_{i}$, the algorithm returns unknown.

#### 4.2.3 Conjunctive template solving.

Conjunctive invariants express multiple properties that must hold simultaneously throughout loop execution, making them essential for verifying complex safety conditions, variable bounds, and structural relationships. Unlike disjunctive invariants that represent alternative execution patterns, conjunctive invariants capture the intersection of constraints that remain preserved across all loops iterations. Formally, a conjunctive invariant takes the form $Inv\equiv C_{1}(\theta_{1})\wedge C_{2}(\theta_{2})\wedge ...\wedge C_{n}(\theta_{n})$, where each clause $C_{i}$ represents an independent property (equality, inequality, interval or extremal constraint) with its own parameters $\theta_{i}$.

Consider the following example where multiple invariant properties must hold concurrently:

assert(n > 0);

int x = 0, y = 0, z = 0;

while (x < n) {

y = y + 2;

z = z + 1;

x++;

}

assert(x == n && y == 2*n && z == n);

Our multi-representation analysis identifies that this loop maintains three simultaneous relationships: (1) *x* progresses linearly toward *n*, (2) *y* increases at twice the rate of *x*, and (3) *z* increments in lockstep with *x*. The AST reveals three independent assignment statements, the DDG shows parallel data dependencies (no clause depends on another), and the CFG confirms a single execution path with no branching. Based on this analysis, our framework constructs a conjunctive template with three clauses:


$$\begin{array}{c} x\leq a_{1}\wedge y=a_{2}\cdot x\wedge z=a_{3}\cdot x \end{array}$$ (5)

where coefficients $\left\{a_{1},a_{2},a_{3}\right\}$ must be instantiated to capture the precise relationships.

**Algorithm 6: Conjunctive Template Solving**



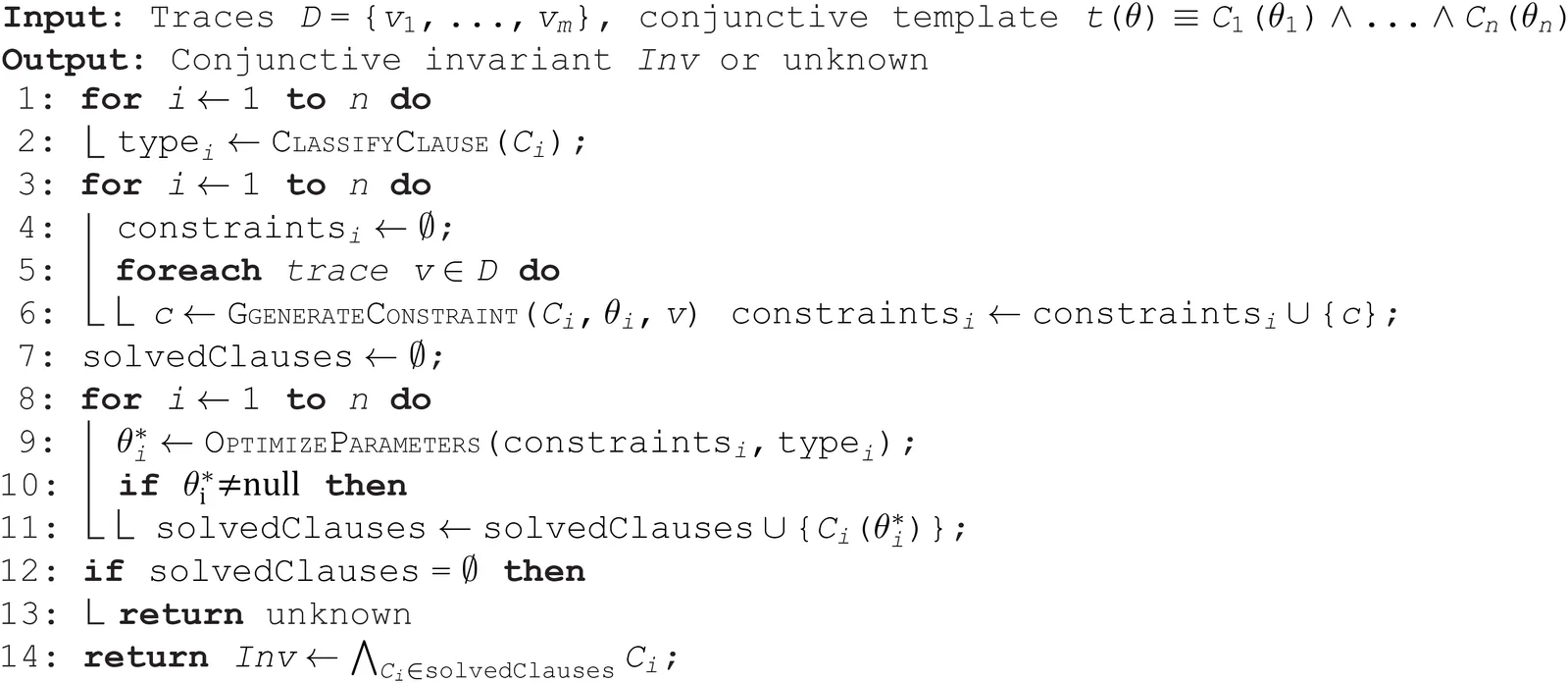



Algorithm 6 presents the procedure for instantiating and solving a conjunctive invariant template using execution traces. The input consists of a set of concrete traces *D* and a conjunctive invariant template t(θ), expressed as a conjunction of parameterized clauses $C_{1}(\theta_{1})\wedge \cdots \wedge C_{n}(\theta_{n})$. The goal of the algorithm is to infer a valid conjunctive invariant by solving each clause independently. The algorithm first classifies each clause (e.g., equality or inequality), which determines the corresponding optimization strategy. For each clause, a set of constraints is generated by instantiating the clause over all execution traces. These constraints characterize the conditions that the clause parameters must satisfy to be consistent with the observed program behaviors. Next, the algorithm independently optimizes the parameters of each clause by solving its associated constraint system. Clauses for which a valid parameter assignment is found are retained, while unsatisfiable clauses are discarded. If no clause can be successfully instantiated, the algorithm returns unknown, indicating that the template cannot be solved with the available traces. Finally, the inferred invariant is constructed as the conjunction of all successfully instantiated clauses.

#### 4.2.4 Invariant soundness guarantees.

In our framework, soundness is guaranteed by the SMT verification phase. Once a candidate invariant *Inv* is produced during template instantiation, it is submitted to the Z3 SMT solver for verification against the three standard correctness conditions. Formally, Z3 checks the negation of each condition as a satisfiability query:

1. **Initialization:** Pre⇒Inv, ensuring that the loop invariant holds immediately before the loop begins execution.
2. **Preservation:** {Inv∧C}ST{Inv}, ensuring that if the invariant and the loop condition *C* both hold, executing the loop body *ST* preserves the invariant for the next iteration.
3. **Termination:** (Inv∧¬C)⇒Post, ensuring that if the loop terminates while the invariant still holds, the final program state satisfies the desired postcondition.

Using the assignment axiom, the verification condition for the loop body reduces to proving: Inv∧C⇒Inv[E/x], where *Inv*[*E*/*x*] denotes the invariant *Inv* with every free occurrence of the variable *x* replaced by the expression *E* [47].Therefore, the framework may return USAT when the instantiated candidate invariant fails to satisfy one or more SMT verification conditions.

## 5 Experimental setting

### 5.1 Benchmark description

We evaluate our framework on a benchmark suite of 100 C loop programs. We primarily draw from existing invariant inference benchmarks [15,48]. However, these benchmarks largely emphasize linear and nonlinear equality invariants, with only limited coverage of disjunctive invariants and more complex forms such as conjunctive and nonlinear inequality invariants. Therefore, our benchmark suite comprises 60 examples selected from these existing benchmarks and 40 newly synthesized programs. The additional 40 programs were generated using a grammar-guided construction process, in which candidate loop bodies were constructed around predefined nonlinear transition relations and multi-variable path conditions and then validated using SMT-based reachability checks to ensure well-defined preconditions and postconditions. This generation procedure was intentionally tailored to introduce underrepresented invariant types, specifically disjunctive, conjunctive and nonlinear inequality invariants. The resulting suite provides a uniform distribution across the six target template classes, reducing classification bias during training.

To facilitate a systematic evaluation of different aspects of our approach, we organize the benchmark into two core groups based on control-flow complexity:

**Single-Path Loops**, the first group consists of 40 single-path loops whose bodies contain no internal branching. Although the control flow within these loops is simple, their syntactic structure and data dependency patterns can vary significantly. These loops typically admit linear or nonlinear polynomial invariants and serve as a foundation for evaluating the model’s ability to identify relevant structural features when the CFG contains a single execution path through the loop body. Representative examples include accumulator loops, counter increments, and arithmetic progression patterns.

**Multi-Path Loops**, the remaining 60 programs are classified in the multi-path group. These benchmarks contain one or more conditional branches within the loop body, resulting in multiple execution paths and more complex control-flow joins. Such programs often require disjunctive reasoning and exhibit intricate data-dependence structures, leading to invariants of substantially higher complexity. This group is significant for evaluating whether the model can effectively integrate information from AST, CFG, and DDG representations to capture subtle interactions between control and data flow.

Table 1 summarizes the distribution and structural characteristics of the 100 benchmark programs across the six invariant template classes. In addition to the number of programs in each class, the table reports the average lines of code (Avg. LOC) and maximum variable count (Max Variables) to reflect program complexity and scalability. The benchmark consists of 40 Single-Path loops and 60 Multi-Path loops, enabling evaluation across varying levels of control-flow complexity. Linear and nonlinear equality/inequality templates primarily appear in simpler arithmetic loops, while conjunctive and disjunctive templates correspond to structurally more complex programs with richer control-flow and data-dependency interactions. In particular, disjunctive invariants appear exclusively in Multi-Path loops and exhibit the highest structural complexity.

**Table 1:** Benchmark Composition and Structural Characteristics Across Invariant Template Classes.

Template Class	Single-Path	Multi-Path	Avg. LOC	Max Variables
Equality linear	10	6	12.4	4
Equality nonlinear	9	8	16.8	6
Inequality linear	9	5	14.1	4
Inequality nonlinear	7	5	15.5	6
Conjunctive	5	6	18.3	8
Disjunctive	0	30	31.4	6
**Total / Summary**	**40**	**60**	**19.1**	**8**

To address the limited size of the benchmark suite, we apply source-level data augmentation to expand the dataset from 100 to 2,000 programs using constant mutation and variable renaming. Constant mutation replaces numeric values within loop bodies, while variable renaming changes identifier names without affecting program semantics. Both transformations preserve the original invariant template class. The dataset is partitioned using Group-*K*-fold cross-validation with *K* = 5, enforced at the base-program level to prevent data leakage between augmented variants. The remaining 20% of the original programs and their augmented variants are reserved as a fixed unseen test set.

### 5.2 Parameter settings

The experiments were conducted on a system equipped with an Intel® Core™ i7 processor and an NVIDIA GeForce RTX 3050 GPU. The implementation was developed using Python 3.9.6, TensorFlow 2.5.0, and Keras 2.5.0. These hardware and software configurations were selected to ensure efficient execution and reproducible results throughout the experimental evaluation. The template construction phase consists of three main sub-processes: program representation, graph embedding, and selection. For program representation, AST, CFG, and DDG are extracted directly from the C source code using Python-based analysis tools. During the embedding stage, AST nodes are represented as numerical vectors using Word2Vec, while CFG and DDG graphs are encoded with Node2Vec to capture structural and semantic relationships. The model of template category selection is implemented in TensorFlow 2.5.0 and Keras 2.5.0. Selected template categories are subsequently solved and checked using the Z3 SMT solver, with a timeout of 30 seconds per candidate template. To ensure robust evaluation, all experiments are repeated 20 times, and results are reported as the mean performance across these runs.

Table 2 summarizes the key hyperparameters used in graph embedding, model training, and template solving. The hyperparameters are determined using three strategies. Node2Vec and Word2Vec parameters follow default settings from the original implementations and prior work on program representation learning [44,45], with embedding dimension set to 100. CNN training hyperparameters (batch size, epochs, and activation functions) were tuned through grid search under the Group-*K*-fold validation scheme described in the previous section. The Z3 [49] solver timeout was selected from {10, 20, 30} seconds using the validation set, with 30 seconds providing the best trade-off between success rate and runtime. The six invariant template categories were defined based on the benchmark to cover all observed invariant types.

**Table 2:** Hyperparameters of the Proposed Approach.

Step	Parameter	Value
Graph Embedding (Node2Vec)	Dimensions	100
	Window size	10
	Batch words	4
	Minimum count	1
Word Embedding (Word2Vec)	Context window size	5
	Vector size	100
	Batch words	50
	Negative sampling	10
	Minimum word frequency	5
category selection Model	Batch size	1024
	Number of epochs	100
	Loss function	Cross-entropy
	Optimizer	Adam
	Activation functions	Sigmoid (fully connected layers)
		Softmax (classification layer)
		ReLU (other layers)
Template Solving	SMT solver	Z3
	Solver timeout	30 seconds per candidate
	Template types considered	equalities and inequalities (Linear and nonlinear),
		Disjunctive and conjunctive

### 5.3 Baseline setting

- *Code2Inv* [48]: A reinforcement learning–based framework that formulates loop invariant inference as a sequential decision-making problem and learns to generate invariants accordingly.
- *CLN2INV* [40]: A deep learning–based approach that establishes a structured correspondence between logical expressions and neural network models. Loop invariants are inferred by training a neural network representation to satisfy constraints derived from program execution traces
- *G-CLN*: [13] An approach extends CLN2INV by introducing gating mechanisms and additional architectural enhancements, allowing it to learn more complex loop invariants. In particular, it demonstrates strong capability in inferring nonlinear loop invariants.
- *Clause2Inv* [41]: A loop invariant inference approach based on a generate–combine–check framework. It uses an LLM-based generator to produce simple clauses and combines them into complex invariants using counterexample-driven refinement.

### 5.4 Evaluation metrics

We assess the performance of our framework using the following metrics, which capture different aspects of template selection accuracy, invariant inference effectiveness, and computational efficiency:

1) **Precision, Recall, and F1-Score** These metrics are used to evaluate the accuracy and coverage of the model at the level of individual template selection. Specifically, we define *TP* as the number of templates correctly selected by the model, *FP* as the number of selected templates that do not match the reference templates, and *FN* as the number of reference templates that the model fails to select. Precision (*P*) measures the correctness of the select templates, recall (*R*) measures the completeness of the selection, and the F1-score (*F*1) captures the balance between precision and recall:

$$\begin{array}{c} R=\frac{TP}{TP+FN},\;P=\frac{TP}{TP+FP},\;F1=\frac{2\cdot P\cdot R}{P+R}. \end{array}$$ (6)

2) **AUC:** The Area Under the ROC Curve (AUC) is computed only for template selection to evaluate the model’s ability to distinguish correct from incorrect templates across varying thresholds.
3) **Invariant Success Rate (ISR)**: the percentage of loops for which our framework successfully generates a valid invariant that passes SMT verification as described in section 4.2.4. This metric evaluates the end-to-end effectiveness of the complete framework, including template construction, template solving, and formal verification. *ISR* is computed as:

$$\begin{array}{c} ISR=\frac{N_{success}}{N_{total}}\times 100 \end{array}$$ (7)

## 6 Result discussion

To evaluate the effectiveness of our proposed approach, we structure our experiments around the following research questions:

- RQ1: Does integrating AST, CFG, and DDG improve invariant template construction compared to using each representation individually?
- RQ2: What is the end-to-end success rate of our framework in generating valid loop invariants?
- RQ3: How does our learning framework compare with state-of-the-art invariant inference tools?

### 6.1 Answer to RQ1: Does integrating AST, CFG, and DDG improve invariant template construction compared to using each representation individually?

We conduct experiments in three stages: AST, AST + DDG, and AST + DDG + CFG, to evaluate the incremental impact of contextual and semantic-graph features. This design follows the hierarchical nature of program analysis, since DDG and CFG cannot be evaluated independently without the AST, which provides the syntactic foundation containing variable identities, constants, and operators required for invariant inference. Accordingly, the first stage captures syntax-only information from the AST, the second adds data-dependency semantics through DDG, and the third further incorporates control-flow information through CFG to measure the cumulative contribution of each semantic layer.

Table 3 presents the Precision (P), Recall (R), F1 scores, and AUC performance of our approach when utilizing different feature sets (AST),(AST + DDG), and (AST + DDG + CFG). The results in Table 3 demonstrate that integrating multiple program representations AST, DDG, and CFG, substantially improves the selection of template category. The AST-only baseline achieves an average *F*1 score of **0.400**, indicating the inherent difficulty of selection invariant template from code syntax alone. Augmenting the model with data dependence information (AST + DDG) increases performance to **0.517** *F*1 (**+11.7%**), while the full model incorporating control flow (AST + DDG + CFG) achieves the best result of **0.588** *F*1 (**+18.8%**). This consistent improvement across all template categories, from simple linear equations to complex disjunctive forms, validates that a holistic, multi-representation approach captures the semantic and contextual relationships necessary for accurate template selection.

**Table 3:** Multi-Representation Impact on Classification Metrics.

Category	AST				AST + DDG				AST + DDG + CFG			
	P	R	F1	AUC	P	R	F1	AUC	P	R	F1	AUC
SL (Eq. (Linear))	.547	.513	.529	.538	.638	.601	.619	.631	.651	.614	.632	.645
SL (Eq. (Nonlinear))	.421	.389	.404	.412	.538	.502	.519	.529	.614	.576	.594	.607
SL (Ineq. (Linear))	.489	.456	.472	.481	.573	.538	.555	.566	.587	.552	.569	.581
SL (Ineq. (Nonlinear))	.358	.331	.344	.351	.487	.453	.469	.479	.581	.544	.562	.574
MLP (Single invariant Eq.(Linear+nonlinear))	.403	.374	.388	.396	.531	.496	.513	.524	.619	.581	.599	.612
MLP (Disjunctive invariant)	.327	.301	.313	.320	.476	.442	.458	.468	.589	.552	.570	.582
nonlinear Conjunctive (MLP + SL)	.369	.341	.354	.362	.502	.468	.484	.494	.607	.569	.587	.600
**Average**	**.416**	**.386**	**.400**	**.409**	**.535**	**.500**	**.517**	**.527**	**.607**	**.570**	**.588**	**.600**
**Improvement**	—				+11.7%				+18.8%			

Fig 4 illustrates the performance metrics for AST (red), AST + DDG (orange), and AST + DDG + CFG (green) across all invariant categories. Subfigures 4b and 4b present the Precision and Recall scores, while Subfigures 4c and 4d present the F1-Scores and AUC values, respectively. Overall, Fig 4 demonstrates that AST + DDG performs better than AST alone, and AST + DDG + CFG performs better than both AST and AST + DDG across all four metrics. The consistent rightward progression of bars in each subfigure confirms that adding data-flow and control-flow representations systematically improves invariant template selection.

**Fig 4:**
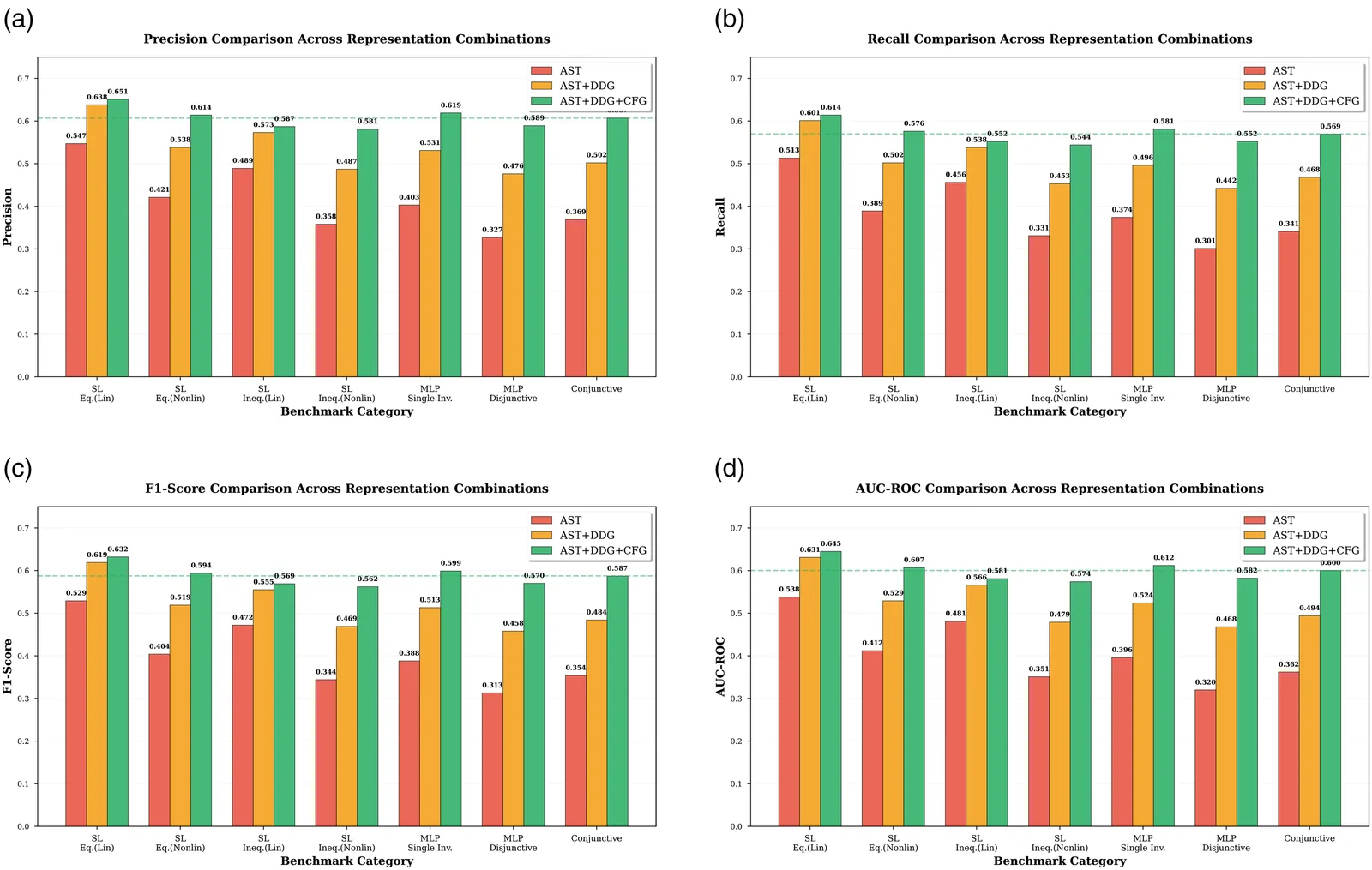
Performance metrics across all benchmark categories for AST (red), AST + DDG (orange), and AST + DDG + CFG (green) representations. The four subplots show (a) Precision, (b) Recall, (c) F1-Score, and (d) AUC.

In summary, the results presented in Table 3 and illustrated in Fig 4 confirm that integrating multiple program representations, syntactic, data and control-flow enhances the accuracy of invariant template category selection across diverse invariant types and complexity levels.

### 6.2 Answer to RQ2: What is the end-to-end success rate of our framework in generating valid loop invariants?

The End-to-end evaluation measures the complete invariant inference pipeline from program input to verified output, assessing whether the framework can automatically generate valid loop invariants without human intervention. It requires all stages of the pipeline, including template construction, constraint solving, and SMT verification, to succeed sequentially for an invariant to be considered valid. This holistic measure reflects practical utility, as failure at any stage prevents the system from producing a sound invariant. Table 4 reports an overall Invariant Success Rate (*ISR*) of 84.0%, with correct invariants generated for 84 out of 100 benchmark cases. Analysis of the 16 failures indicates that template selection is the dominant source of failure, accounting for 68.75% of all failures (11/16). Most misclassifications occur between structurally similar categories, particularly equality_nonlinear and inequality_nonlinear, as well as disjunctive and conjunctive. These ambiguities contribute to the lower ISR observed in the *MLP (Disjunctive Invariant)* category, where control-flow distinctions are less explicitly represented in the learned embeddings. The remaining failures arise during SMT verification, accounting for 31.25% of all failures (5/16). In these cases, candidate invariants satisfy the observed execution traces but fail the formal preservation conditions over all reachable states. While these failures highlight limitations in trace coverage, they also demonstrate that the SMT verification stage effectively preserves soundness by rejecting invalid invariants rather than producing unsound results.

**Table 4:** Invariant Success Rate (ISR) by Benchmark Category.

Category	*N*_total_	*N*_success_	ISR (%)
**Single-Path (*N* = 40)**			
Linear Equality	10	10	100
Nonlinear Equality	9	8	100
Linear Inequality	9	8	88.9
Nonlinear Inequality	7	5	71.4
Conjunctive Invariant	5	4	80.0
**Multi-Path (*N* = 60)**			
Linear Equality	6	6	100
Nonlinear Equality	8	7	87.5
Linear Inequality	5	4	80.0
Nonlinear Inequality	5	3	60.0
Conjunctive Invariant	6	5	83.3
Disjunctive Invariant	30	23	76.7
**Overall**	**100**	**84**	**84.0**

### 6.3 Answer to RQ3: How does our learning framework compare with state-of-the-art invariant inference tools?

Table 5 presents a comparison of our framework against four state-of-the-art baselines (CLN2INV, Code2Inv, Clause2Inv, and G-CLN) in terms of template selection strategy, template expressiveness, and Invariant Success Rate (ISR). Unlike existing methods, which rely on manually predefined templates and thus require users to specify the invariant structure a priori, our framework automatically predicts the appropriate template category using a learning-based approach that operates over joint AST, CFG, and DDG representations. This enables fully automated invariant inference without human template engineering. In contrast, CLN2INV and Code2Inv are restricted to linear forms and lack nonlinear support. Clause2Inv extends expressiveness through LLM-based generation but still depends on predefined structures, and G-CLN offers broader coverage yet remains limited by manual specification and difficulty handling disjunctive invariants. As a result, our method achieves the highest ISR (84.0%), outperforming CLN2INV (42.0%), Code2Inv (51.0%), Clause2Inv (63.0%), and G-CLN (68.0%), while also supporting the most comprehensive template space (linear/nonlinear equalities and inequalities, as well as conjunctive and disjunctive invariants), demonstrating that learning-based template selection with rich program representations leads to more accurate and fully automated invariant synthesis.

**Table 5:** Quantitative Performance Comparison Against Baselines.

Method	Learning Paradigm	Supported Template catogerySS	ISR (%)
CLN2INV	CLN	Linear Equalities Only	42.0%
Code2Inv	RL + GNN	Simple Disjunctive & Linear Equalities (No Nonlinear)	51.0%
Clause2Inv	LLM + Counterexample	Linear & Nonlinear Equalities	63.0%
G-CLN	Gated CLN	Linear/Nonlinear Equalities & Inequalities & Conjunctive	68.0%
**Our Framework**	**CNN**	**Linear/Nonlinear, Disjunctive/Conjunctive**	**84.0%**

## 7 Threats to validity

We identify several threats to the validity of our experimental evaluation, primarily related to external validity and scalability. A potential threat to external validity arises from the benchmark suite used in this approach, which may not fully capture the diversity of loop structures and invariant patterns encountered in real-world software systems. In particular, programs whose invariants fall outside the six template categories supported by our framework are inherently underrepresented. Consequently, the reported performance may not generalize to arbitrary benchmarks containing substantially different invariant forms or highly domain-specific behaviors.

Another threat concerns scalability. The current evaluation focuses primarily on programs involving a moderate number of variables and polynomial invariants of degree three or lower. As the number of variables increases or higher-degree polynomial relations are introduced, the invariant search space grows combinatorially, creating additional challenges for both template instantiation and SMT verification. Although the proposed multi-representation graph stacking mitigates part of this complexity by providing precise structural guidance during template selection, further evaluation on large-scale software systems with high-dimensional variable spaces and more complex polynomial behaviors is necessary to fully assess the scalability and robustness of the approach.

## 8 Conclusion

This paper presented a deep learning–based framework for automated loop invariant inference that integrates multiple complementary program representations to capture both the syntactic and semantic characteristics of program loops. The proposed framework combines AST, DDG, and CFG to provide a more comprehensive representation of loop behavior. The framework follows a two-phase architecture consisting of invariant template selection and template instantiation. In the first phase, a structure-aware CNN predicts the most suitable invariant template category directly from program structure. In the second phase, the selected template is instantiated using execution traces to infer parameter values, producing a concrete invariant candidate that is subsequently validated using an SMT solver.

Experimental evaluation on 100 benchmark programs demonstrates that the proposed framework achieves an Invariant Success Rate *ISR* of 84.0%, indicating strong effectiveness in fully automated invariant inference. By jointly leveraging syntax, control flow, and data-dependence information, the framework effectively handles loops involving nonlinear arithmetic, complex branching behavior, and multiple execution paths. Compared with traditional template-based approaches that rely on manually predefined invariant structures, the proposed learning-guided framework significantly improves automation while maintaining structured and formally verified invariant synthesis. Future work will focus on extending support to additional invariant categories, improving robustness for more complex program behaviors, and enhancing scalability to larger software systems with higher-dimensional variable spaces and more complex polynomial relations

## References

[1] Grimm T, Lettnin D, Hübner M. A Survey on Formal Verification Techniques for Safety-Critical Systems-on-Chip. Electronics. 2018;7(6):81. doi: 10.3390/electronics7060081

[2] Gao M, Wang X, Zhu X. The Verification Technology for Aerospace Embedded Software Safety. Aerospace Control. 2025;43:72–8.

[3] Furia CA, Meyer B, Velder S. Loop invariants: analysis, classification, and examples. ACM Comput Surv. 2014;46:1–51.

[4] Hoare CAR. Proof of a program: FIND. Commun ACM. 1971;14(1):39–45. doi: 10.1145/362452.362489

[5] Cousot P, Cousot R. Abstract Interpretation Frameworks. J Logic Computat. 1992;2(4):511–47. doi: 10.1093/logcom/2.4.511

[6] Cousot P. Abstract interpretation. ACM Comput Surv. 1996;28:324–8.

[7] McMillan KL. Interpolation and SAT-Based Model Checking. In: Computer Aided Verification: 15th International Conference, CAV 2003, Boulder, CO, USA, July 8–12, 2003. Proceedings. 2003. p. 1–13.

[8] McMillan KL. Lazy abstraction with interpolants. In: Computer aided verification: 18th international conference, CAV 2006, Seattle, WA, USA, August 17–20, 2006. Proceedings 18. 2006. p. 123–36.

[9] Ernst MD, Cockrell J, Griswold WG, Notkin D. Dynamically discovering likely program invariants to support program evolution. In: Proceedings of the 21st international conference on Software engineering. 1999. p. 213–24.

[10] Ernst MD. Dynamically Detecting Likely Program Invariants. University of Washington; 2000.

[11] Ernst MD, Perkins JH, Guo PJ, McCamant S, Pacheco C, Tschantz MS, et al. The Daikon system for dynamic detection of likely invariants. Sci Comput Programm. 2007;69(1–3):35–45. doi: 10.1016/j.scico.2007.01.015

[12] Nguyen T, Dwyer MB, Visser W. Symlnfer: Inferring program invariants using symbolic states. In: 2017 32nd IEEE/ACM International Conference on Automated Software Engineering (ASE). 2017. p. 804–14.

[13] Yao J, Ryan G, Wong J, Jana S, Gu R. Learning nonlinear loop invariants with gated continuous logic networks. In: Proceedings of the 41st ACM SIGPLAN Conference on Programming Language Design and Implementation. 2020. p. 106–20.

[14] Xu R, He F, Wang B-Y. Interval counterexamples for loop invariant learning. In: Proceedings of the 28th ACM Joint Meeting on European Software Engineering Conference and Symposium on the Foundations of Software Engineering. 2020. p. 111–22.

[15] Nguyen T, Nguyen K, Dwyer MB. Using Symbolic States to Infer Numerical Invariants. IIEEE Trans Software Eng. 2021;48(10):3877–99. doi: 10.1109/tse.2021.3106964

[16] Yu S, Wang T, Wang J. Loop Invariant Inference through SMT Solving Enhanced Reinforcement Learning. In: Proceedings of the 32nd ACM SIGSOFT International Symposium on Software Testing and Analysis. 2023. p. 175–87.

[17] Sharma R, Gupta S, Hariharan B, Aiken A, Liang P, Nori AV. A Data Driven Approach for Algebraic Loop Invariants. In: European Symposium on Programming. 2013. p. 574–92.

[18] Sharma R, Aiken A. From invariant checking to invariant inference using randomized search. Form Methods Syst Des. 2016;48(3):235–56. doi: 10.1007/s10703-016-0248-5

[19] Garg P, Löding C, Madhusudan P, Neider D. ICE: A Robust Framework for Learning Invariants. In: International Conference on Computer Aided Verification. 2014. p. 69–87.

[20] Garg P, Neider D, Madhusudan P, Roth D. Learning invariants using decision trees and implication counterexamples. SIGPLAN Not. 2016;51(1):499–512. doi: 10.1145/2914770.2837664

[21] Si X, Dai H, Raghothaman M, Naik M, Song L. Learning loop invariants for program verification. Adv Neural Inform Process Syst. 2018;31.

[22] Colón MA, Sankaranarayanan S, Sipma HB. Linear Invariant Generation Using Non-Linear Constraint Solving. In: Computer Aided Verification: 15th International Conference, CAV 2003. 2003. p. 420–32.

[23] Gupta A, Majumdar R, Rybalchenko A. From Tests to Proofs. In: International Conference on Tools and Algorithms for the Construction and Analysis of Systems. 2009. p. 262–76.

[24] Cousot P, Cousot R. Systematic Design of Program Analysis Frameworks. In: Proceedings of the 6th ACM SIGACT-SIGPLAN Symposium on Principles of Programming Languages. 1979. p. 269–282.

[25] Cousot P, Halbwachs N. Automatic Discovery of Linear Restraints among Variables of a Program. In: Proceedings of the 5th ACM SIGACT-SIGPLAN Symposium on Principles of Programming Languages. 1978. p. 84–96.

[26] Karr M. Affine relationships among variables of a program. Acta Inform. 1976;6(2):133–51. doi: 10.1007/bf00268497

[27] Calcagno C, Distefano D, Vafeiadis V. Bi-Abductive Resource Invariant Synthesis. In: Asian Symposium on Programming Languages and Systems. 2009. 259–74.

[28] Dillig I, Dillig T, Li B, McMillan K. Inductive Invariant Generation via Abductive Inference. ACM SIGPLAN Not. 2013;48:443–56.

[29] Hojjat H, Rümmer P. The ELDARICA Horn Solver. In: 2018 Formal Methods in Computer Aided Design (FMCAD). 2018. p. 1–7.

[30] Vediramana Krishnan HG, Chen Y, Shoham S, Gurfinkel A. Global guidance for local generalization in model checking. Form Methods Syst Des. 2024;63(1–3):81–109. doi: 10.1007/s10703-023-00412-3 39678253 PMC11639202

[31] Jhala R, McMillan KL. A Practical and Complete Approach to Predicate Refinement. In: International Conference on Tools and Algorithms for the Construction and Analysis of Systems. 2006. p. 459–73.

[32] McMillan KL. Lazy Annotation for Program Testing and Verification. In: International Conference on Computer Aided Verification. 2010. p. 104–18.

[33] Barbosa H, Barrett C, Brain M, Kremer G, Lachnitt H, Mann M, et al. cvc5: A Versatile and Industrial-Strength SMT Solver. In: International Conference on Tools and Algorithms for the Construction and Analysis of Systems. 2022. p. 415–42.

[34] Barrett C, Conway CL, Deters M, Hadarean L, Jovanović D, King T, et al. CVC4. In: International Conference on Computer Aided Verification. 2011. p. 171–7.

[35] Clarke E, Grumberg O, Jha S, Lu Y, Veith H. Counterexample-guided abstraction refinement for symbolic model checking. J ACM. 2003;50(5):752–94. doi: 10.1145/876638.876643

[36] Zhu H, Nori AV, Jagannathan S. Learning refinement types. SIGPLAN Not. 2015;50(9):400–11. doi: 10.1145/2858949.2784766

[37] Farzan A, Kincaid Z. Compositional Recurrence Analysis. In: 2015 Formal Methods in Computer-Aided Design (FMCAD). 2015. p. 57–64.

[38] Humenberger A, Jaroschek M, Kovács L. Invariant Generation for Multi-Path Loops with Polynomial Assignments. In: International Conference on Verification, Model Checking, and Abstract Interpretation. 2017. p. 226–246.

[39] Kincaid Z, Cyphert J, Breck J, Reps T. Non-linear reasoning for invariant synthesis. Proc ACM Program Lang. 2017;2(POPL):1–33. doi: 10.1145/3158142

[40] Ryan G, Wong J, Yao J, Gu R, Jana S. CLN2INV: Learning Loop Invariants with Continuous Logic Networks. arXiv preprint. 2019;arXiv:1909.11542.

[41] Cao W, Wu G, Xu T, Yao Y, Wei H, Chen T, et al. Clause2Inv: A Generate-Combine-Check Framework for Loop Invariant Inference. Proc ACM Softw Eng. 2025;2(ISSTA):1009–30. doi: 10.1145/3728920

[42] Aho AV, Lam MS, Sethi R, Ullman JD. Compilers: Principles, Techniques, & Tools. 2nd edition. Pearson Education; 2007.

[43] Harrold MJ, Soffa ML. Efficient computation of interprocedural definition-use chains. ACM Trans Program Lang Syst. 1994;16(2):175–204. doi: 10.1145/174662.174663

[44] Mikolov T, Sutskever I, Chen K, Corrado GS, Dean J. Distributed Representations of Words and Phrases and Their Compositionality. In: Advances in Neural Information Processing Systems, 2013.

[45] Grover A, Leskovec J. node2vec: Scalable Feature Learning for Networks. In: Proceedings of the 22nd ACM SIGKDD International Conference on Knowledge Discovery and Data Mining. 2016. p. 855–64. 10.1145/2939672.2939754 PMC5108654 27853626

[46] Kingma DP, Ba J. Adam: A Method for Stochastic Optimization. In: International Conference on Learning Representations (ICLR). 2015.

[47] Hoare CAR. An Axiomatic Basis for Computer Programming. Commun ACM. 1969;12(10):576–80.

[48] Si X, Naik A, Dai H, Naik M, Song L. Code2Inv: A Deep Learning Framework for Program Verification. In: International Conference on Computer Aided Verification. 2020. p. 151–64.

[49] De Moura L, Bjørner N. Z3: An Efficient SMT Solver. In: International Conference on Tools and Algorithms for the Construction and Analysis of Systems. 2008. p. 337–40.

